# Is it safe and efficacious to remove central lines in pediatric bone marrow transplant patients with platelets less than 20,000/μl?

**DOI:** 10.1002/jha2.379

**Published:** 2022-01-15

**Authors:** Priya Marwah, Stalin Ramprakash, Sai Prasad T R, Mane Gizhlaryan, Deepa Trivedi, Vaibhav Shah, Amit Chitaliya, Sandeep Elizabeth, Rajat Kumar Agarwal, Rakesh Dhanya, Lawrence Faulkner

**Affiliations:** ^1^ Department of Pediatrics Mahatma Gandhi University of Medical Sciences and Technology Jaipur India; ^2^ Sankalp India Foundation Bangalore India; ^3^ Sankalp‐People Tree Centre for Paediatric Bone Marrow Transplantation Bangalore India; ^4^ Sankalp‐CIMS Centre for Paediatric BMT Ahmedabad India; ^5^ Jagriti InnoHealth Platforms Pvt. Ltd. Bangalore India; ^6^ Cure2Children Foundation Florence Italy

**Keywords:** antimicrobial stewardship, bone marrow transplantation, central venous catheter, febrile neutropenia, thrombocytopenia

## Abstract

**Background:**

Patients with tunneled central venous lines (CVL) may develop bloodstream infections which at times are difficult to control without line removal. Concomitant severe thrombocytopenia with platelet transfusion refractoriness is often considered a major contraindication to any procedure involving a major blood vessel. There is very little literature on the clinical risks of tunneled central line removal in febrile pancytopenia patients.

**Procedure:**

We analyzed complications and outcomes in all our patients, a total of 52, who underwent CVL removal with platelets <20,000/μl.

**Results:**

CVL removal was done on a median day of 17.5 with 47 of the 52 patients never having achieved platelets engraftment prior to line removal. No bleeding episodes or unplanned transfusions could be associated with CVL removal. No other complications were also reported. All patients had time to hemostasis within 5 min of catheter removal. Removal of CVL under local anesthesia remained complication‐free even at platelet counts less than 20,000/ul. A total of 31 patients were febrile at the time of CVL removal, of which 17 became afebrile within 2 days. We found no difference in defervescence when comparing those whose antibiotic therapy was changed/escalated versus those in whom it was not.

**Conclusion:**

Our findings suggest that central lines can be safely removed with platelet counts less than 20,000/ul and that this may result in enhanced bloodstream infection control. This might be particularly relevant to neutropenic patients in this day and age of multidrug‐resistant organism emergence and paucity of new effective antibiotics.

**ABBREVIATION KEY**
CIMS: Sankalp‐CIMS Centre for Pediatric BMT, Ahmedabad
CRP: C‐reactive protein
CVL: Central venous lines
HCT: hematopoetic stem cell transplantation
PCT: procalcitonin
PTH: sankalp‐People Tree Centre for Pediatric BMT, Bangalore

## 1 BACKGROUND

Indwelling central venous lines (CVL) may develop biofilms and microthrombi, protecting organisms from antibiotics [1]. As a result, patients with tunnelled CVL may develop bloodstream infections which at times are difficult to control without line removal, particularly if neutropenic. Concomitant severe thrombocytopenia with platelet transfusion refractoriness is often considered a major contraindication to any procedure involving a major blood vessel. There is however very little literature on the actual clinical risks of tunnelled central line removal in febrile pancytopenic patients not responding to second‐line broad‐spectrum parenteral antibiotic therapy, while the safety of USG‐guided central line insertion in thrombocytopenic patients is well established [2, 3, 4].

We analyzed complications and outcomes in all or patients, a total of 52, who underwent CVL removal with platelets <20,000/μl.

## 2 PATIENTS AND METHODS

The medical records available on BMTPlus [5] of all 294 patients who underwent a total of 302 transplants at the Sankalp‐People Tree Centre for Pediatric BMT, Bangalore (PTH) and Sankalp‐CIMS Centre for Pediatric BMT, Ahmedabad (CIMS), between August 2015 and March 2020 were analysed to identify patients who had CVL removed while having a baseline platelet count less than 20,000/μl, which identified 52 such instances. Baseline platelets count was obtained on the day of removal prior to platelets infusions. Of these 52 transplants 31 were boys and 21 were girls with a median age 10.1 (IQR 7.9–13.0) years. Indication for transplant was severe thalassemia for 46 patients (3 splenectomised), severe aplastic anaemia for four patients, Fanconi's anaemia for one patient and sickle cell disease for one patient.

Blood counts, transfusions, maximum daily temperature, clinical notes, culture reports, C‐reactive protein (CRP), Procalcitonin (PCT) and antibiotic therapy were extracted from patient records for 2 days prior to 2 days after CVL removal.

Patients who had a haemoglobin drop of more than 1 gm/dl on the day after line removal were identified. Defervescence (maximal daily temperature less than 38°C) within 48 h of line removal was the primary outcome sought. Antibiotics orders were reviewed looking for change on the same day as line removal. Any drop in CRP and PCT values post line removal was also examined for those who had CRP greater than 0.6 mg/dl and PCT greater than 0.5 ng/ml prior to line removal. Culture reports were examined to identify any evidence of central line infection. Clinical notes were reviewed to identify any complication related to central line removal.

CVL was removed at the bedside under local anaesthesia at PTH [5] while sedation was employed at CIMS hospital [6].

Fisher's exact test was performed using R version 3.5.x to see if there was a difference between those who had an escalation/change in antibiotics versus those who did not at the time of line removal.

## 3 RESULTS

### 3.1 Safety

**FIGURE 1:**
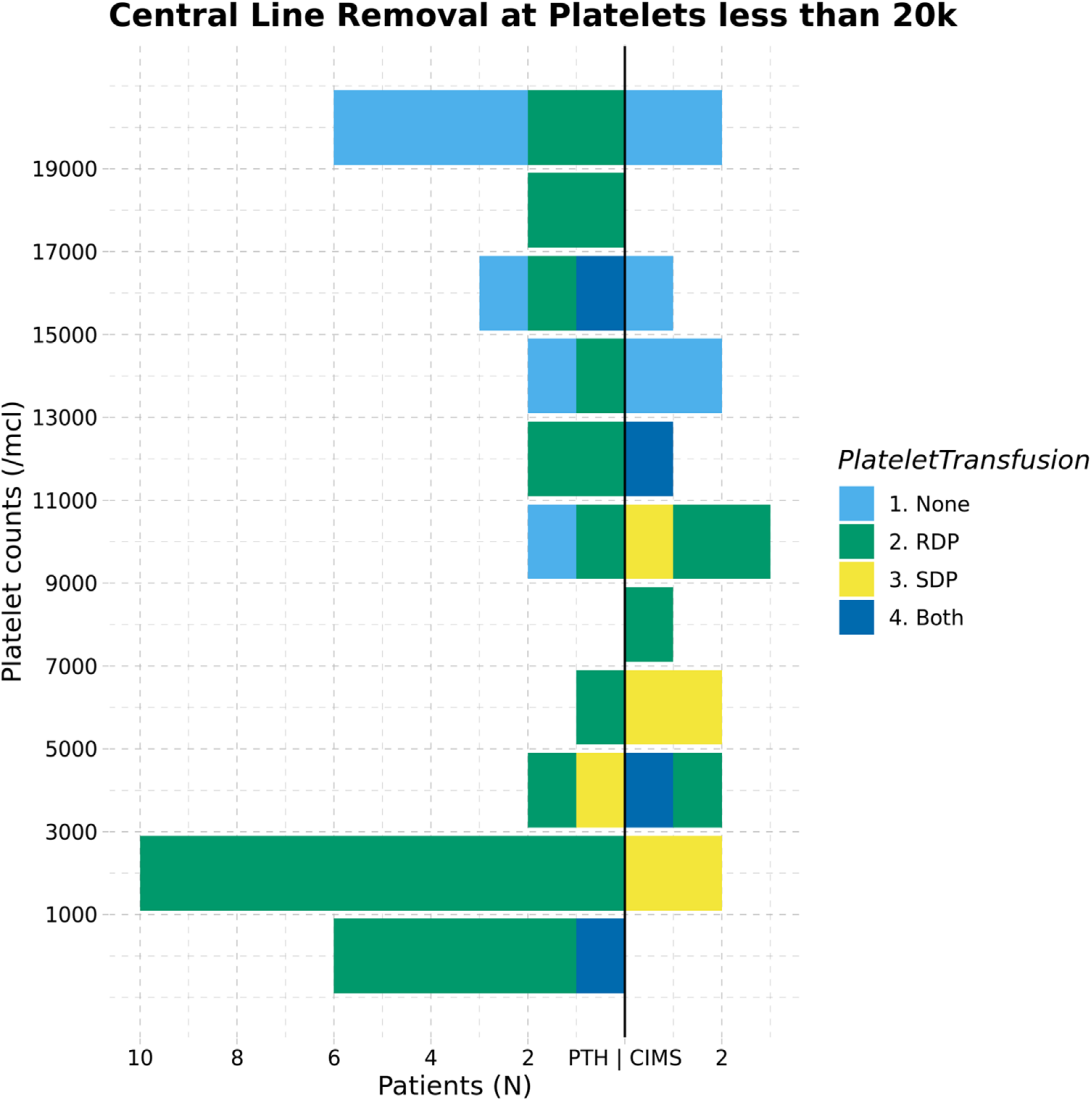
Central line removal at platelets less than 20,000/μl

Central lines were removed from the 52 patients with platelets less than 20,000/μl on a median day 17.5 (interquartile range: 13—26.3) post transplantation. All patients had received platelet transfusions through their transplant course prior to the day of line removal. Platelets had not yet engrafted in 47 patients while the remaining had a decrease in platelet counts post engraftment at the time of line removal. Only random donor platelets were transfused in 30 patients, single donor platelets alone were infused in six patients, both were used in five patients and 11 patients had no platelets transfusion on the day of line removal. In all, nine patients who had a haemoglobin less than 8 gm/dl received packed red cell transfusions on the day of CVL removal. A haemoglobin drop of more than 1gm/dl was observed in five patients however none of them had any overt bleeding. No bleeding episodes or unplanned transfusions could be associated with CVL removal. No other complications were also reported. Table 1 summarises transfusions and significant bleeding. Figure 1 shows the distribution of central line removal at platelets less than 20,000/μl at each of the centre along with the nature of platelet transfusion given.

**TABLE 1:** Total central venous lines (CVL) removals, platelets transfusion, and complications versus platelets range for PTH and CIMS, respectively

	**CIMS**			**PTH**			
	**All**	**0–10k**	**10–20k**	**All**	**0–10k**	**10–20k**	**Total**
Total transplants	**116**			**186**			**302**
CVLs removed <20,000	**16**	8	8	**36**	19	17	**52**
PRBC transfused	**2**	1	1	**7**	6	1	**9**
Hb Drop >1 g/dl	**2**	1	1	**3**	1	2	**5**
Bleeding post CVL removal or unplanned RBC transfusions	**0**	0	0	**0**	0	0	**0**
Other CVL removal‐related complications	**0**	0	0	**0**	0	0	**0**
Platelets on next day of CVL removal 20,000	**13**	7	6	**26**	19	7	**39**

### 3.2 Efficacy

A total 31 patients were febrile at the time of CVL removal, of which 17 became afebrile within 2 days, 12 continued to have fever and two died. Both the patients who died had neutropenic sepsis and one also had steroid refractory graft versus host disease (GVHD). These deaths were not associated with the possible complications of line removal. Among the 29 patients whose fever response could be evaluated, antibiotics were changed or escalated in 16 of the patients together with line removal. Among these, 10 became afebrile. Of the 13 patients who had no antibiotics escalation, seven became afebrile. Fisher's exact test performed to compare those whose antibiotic therapy was changed/escalated versus those in whom it was not, showed no difference in response (*p* = 0.7). Positive blood cultures from CVL sampling were found in 16 patients of whom seven became afebrile within 2 days of line removal. Table 2 summarises the fever and infections status.

**TABLE 2:** Summary of the fever and infections status

	**Fever at CVL removal**	**Fever at CVL removal and no antibiotics change**	**Positive CVL cultures**
**CIMS**	**7**	**3**	**7**
Response	4	2	3
No response	3	1	4
**PTH**	**24**	**10**	**9**
Response	13	5	4
No response	9	5	4
Died prior to response	2		1
**Total**	**31**	**13**	**16**

Only at one of the two centres enough CRP and PCT reports were available to be able to assess a response to CVL removal. Of the 18 patients with elevated CRP at CVL removal and follow‐up values, three showed decrease within 2 days. Of the five patients with elevated PCT levels and follow‐up values, two showed a decrease within 2 days. Table 3 summarises the CRP and PCT response to line removal.

**TABLE 3:** Summary of the C‐reactive protein (CRP) and Procalcitonin (PCT) response to line removal

	**CRP**	**PCT**
**CIMS**		
Not elevated	–	
Response	–	
No response	–	
Undetermined/Unknown	16	16
**PTH**		
Not elevated	3	3
Response	3	2
No response	15	3
Undetermined/Unknown	15	28

## 4 DISCUSSION

Haematopoietic stem cell transplantation (HCT) is associated with variable periods of severe thrombocytopenia. However, major bleeding (defined as any bleeding other than petechiae or mucosal) occurs in a minority of patients and it is unclear if the administration of platelets is required for central venous catheter removal.

Our findings were similar to those observed by Stecker et al [7]. To minimize local trauma while removing the CVL we did not make an extra effort to remove the polyester cuff if it separated from the catheter as this has been shown to be of no clinical significance in most patients [8]. Moreover, in our subset of patients, primarily with non‐malignant diseases, the tunnelled CVL was inserted at the time of conditioning initiation, so that little or no cuff fibrosis was present at the time of removal. One concern may be the achievement of haemostasis if traction removal fails in a particular patient and a cut down is required to remove the catheter. Unlike the study by Stecker et al [7], none of our patients developed this complication. Interestingly, only about five minutes of manual compression were needed to attain complete haemostasis.

Bedside removal of CVL under local anaesthesia remained complication‐free even at platelet counts less than 20,000/μl. Haemoglobin drop of more than 1 g/dl was observed in five of the patients but none showed any signs of overt bleeding and did not require any packed red blood cells (PRBC) transfusion post CVL removal.

Although Stecker et al [7] in their study reported adverse events like bruising, minimal blood oozing, and discomfort, which are not uncommonly seen, none of our cases reported any of these events.

Even if the mortality associated with CVL infections is still a subject of methodological debates [9, 10], morbidity is well documented and includes severe sepsis and septic shock, septic thrombophlebitis, endocarditis and thromboembolism [11]. Neutropenia is a major independent risk factor for CVL infections, and neutropenic patients with bloodstream infections are at a higher risk of mortality compared with non‐neutropenic patients [12]. In our cases, defervescence and septic markers response was independent of concomitant neutrophil recovery.

Although patients undergoing allogeneic or autologous HCT are commonly neutropenic, transplantation might further increase the risk of CVL infections independent of the impact of neutropenia. In a recent retrospective study by McDonald and colleagues, on 352 patients undergoing allogeneic HCT, the use of a matched unrelated donor (MUD) and/or haploidentical donor and the use of an ablative conditioning regimen were independently associated with the development of CVL infections on multivariate analysis [13].

The emergence of multidrug resistance (MDR) organisms is a growing threat [14] so that any measure limiting the prolonged use of high‐end antibiotics is particularly relevant, and CVL removal becomes a necessity to reduce morbidity and eventually mortality in patients at risk of hospital‐acquired infections.

Placement of tunnelled central venous catheters has been extensively studied, but we were not able to find any reports on removal‐related complications during severe pancytopenia or the impact of PT, INR, aPTT or platelets transfusions before traction catheter removal.

In conclusion, though our study has limitations in its sample size, it suggests that central lines can be safely removed with platelet counts less than 20,000/μl and that this may result in enhanced bloodstream infection control. This might be particularly relevant to neutropenic patients in this day and age of MDR organisms emergence and paucity of new effective antibiotics. In our opinion, the risk of infection progression leaving an indwelling CVL in pancytopenia patients with persistent fever not responding to broad‐spectrum antibiotics far outweigh the minimal risk of severe bleeding associated with CVL removal during severe thrombocytopenia.

## AUTHOR CONTRIBUTIONS

PM, MG RD, RKA, and LF wrote the manuscript. SR, SP, DT, VS, AC, and LF participated in medical management. RD, RKA, and LF were involved in conception, design, acquisition, analysis, or interpretation of data. All authors critically reviewed and approved the manuscript.

## CONFLICT OF INTEREST

The authors declare no conflict of interest.
